# Recent Progress in Solid‐State Lithium Batteries through Cathode Microstructure Engineering

**DOI:** 10.1002/advs.202513455

**Published:** 2025-11-19

**Authors:** Hyunji Park, Samuel David Miller, Guanyi Wang, Yue Feng, Jianlin Li, Yuepeng Zhang, Zhenxing Feng

**Affiliations:** ^1^ Applied Materials Division Argonne National Laboratory Lemont IL 60439 USA; ^2^ School of Chemical Biological, and Environmental Engineering Oregon State University Corvallis OR 97331 USA

**Keywords:** cathode engineering, composite cathodes, interface, microstructure, solid‐state batteries

## Abstract

A high‐performance cathode is necessary to realize the great potentials of solid‐state batteries such as high energy density and long cycle life. It is also needed to validate electrolyte performance, which is lacking. Currently, cathodes for solid‐state batteries are thinner and have lower cathode active material content than their lithium‐ion battery counterpart, resulting from insufficient conductivity and limiting the battery energy density. This review article provides an overview of recent development in cathode microstructures and their impact on battery properties, including compatibility between cathode and electrolyte, cathode architecture design, interface engineering, correlation between material properties, cathode processing approaches, and performance, as well as the advanced characterization methods used to understand the above correlations . Some perspectives on future development are shared including utilizing in situ and operando characterization tools to better understand dynamic evolution of the cathode/electrolyte interface, adapting artificial intelligence and machine learning to design and optimize cathode structures. The article is aimed to promote research interest on cathode development and advance solid‐state battery technologies.

## Graphical abstract

Solid‐state batteries offer transformative advancements in energy density and safety, yet they are still in the early stages of development and face significant technical challenges. Here recent advancements in cathode are explored emphasizing the challenges of developing efficient cathode/electrolyte interfaces. It delves into advancements in coating techniques, composition adjustment, cathode processing, and structural design methodologies.

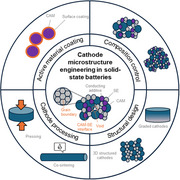

## 1 Introduction

Solid‐state batteries (SSBs) are considered as a “next‐generation” battery technology, offering several compelling advantages over traditional lithium (Li)‐ion batteries (LIBs). Among all the advantages, higher energy density and enhanced safety stand out as particularly significant merits. SSBs can potentially deliver higher energy density, attributed to the replacement of graphite with higher‐capacity lithium, higher operation voltage, and a more compact battery design compared to LIBs.^[^ ^1^ ^]^ Enhanced safety is enabled by eliminating flammable liquid electrolytes (LEs), albeit some chemistries, such as sulfur cathode and polysulfide electrolytes, still pose safety hazards.

Despite their great potential, SSBs are still in an early stage of development and face many challenges. One major challenge is to develop an electrolyte that meets all the requirements for SSBs including high Li‐ion conductivity, low electronic conductivity, and high mechanical, chemical, and electrochemical stability. Additionally, the electrolyte must be easy to process and cost‐effective.^[^ ^2^ ^]^ Extensive research has been conducted on electrolyte development, and several types of solid‐state electrolytes have been reported. However, these electrolytes still suffer from various drawbacks and only meet some of the requirements.^[^ ^3^ ^]^ Beyond material properties, the processing and manufacturing of electrolytes are nontrivial. Significant differences in various electrolytes make it impossible to develop a universal process applicable to all types. Most SSBs in literature so far have thick electrolytes and thin cathodes, resulting in much lower energy density than state‐of‐the‐art LIBs.^[^ ^4^ ^]^ Electrolytes can achieve sufficient conductivity; however, producing thin electrolytes with minimal defects is critical to maintain high conductivity. Unlike LIBs, where liquid electrolytes wet the electrodes and Li‐ions diffuse through a pore network, Li‐ion diffusion in SSBs occurs through the solid phase. Any defects, such as pores, impurities, inhomogeneities, or cracks, are detrimental to Li‐ion diffusion. To this end, material processing and electrode engineering are of utmost importance.

Herein, we review recent progress in cathode development for SSBs. In general, engineering an intimate interface between the cathode and electrolyte has been a daunting challenge for SSB cathodes.^[^ ^5^ ^]^ To ensure high energy density, the amount of catholyte needs to be minimized. It is necessary to fabricate cathodes with a high active material content (e.g., >90 wt%) and high areal capacity (e.g., >3 mAh cm^−2^). Unlike liquid electrolytes, which can conform to electrode surfaces, solid electrolytes (SEs) and cathode materials have limited point contact, resulting in a small contact area. Volume expansion and shrinkage during cycling present additional challenges, potentially leading to cracks and disconnection between cathodes and electrolytes, thereby a high interfacial resistance. As cathode manufacturing processes are specific to particular electrolytes and multiple types of electrolytes exist, here we focus on cathodes with oxide and polymer electrolytes. Other electrolytes will only be briefly mentioned. This review particularly covers electrode microstructure and architecture, interface engineering, characterization and testing. Remaining challenges and potential approaches are discussed at the end.

## 2 Materials, Cell Structures, and Characterization

### 2.1 Materials Comprising Composite Cathode

With an emphasis on manufacturing and design strategies aimed at improving performance and addressing current challenges in SSBs, this section provides a general overview of commonly used materials in SSBs, along with a brief discussion of their merits.

#### 2.1.1 Solid Electrolytes

The SE is a critical component of SSBs acting as both an ion conductor and a physical separator between the cathode and anode. To effectively serve these roles, four main characteristics are of interest: 1) high ionic conductivity, 2) high mechanical strength, 3) chemical and reduction–oxidation (redox) stability, and 4) thermal stability.^[^ ^6^ ^]^ High ionic conductivity is necessary for fast Li‐ion transport allowing for a high charge and discharge capacity to be realized. High mechanical strength serves to keep the electrolyte layer intact when experiencing volume changes, preventing dendrite penetration through the electrolyte layer and improving processability. Chemical and redox stability with both the cathode and anode prevent any detrimental side reactions that lead to greater internal resistance and reduction in capacity over time. Lastly, thermal stability is essential for the overall safety of the SSB by reducing chances of thermal runaway and ensuring more consistent ionic conductivity across a wider temperature range.^[^ ^7^ ^]^

**Figure 1:**
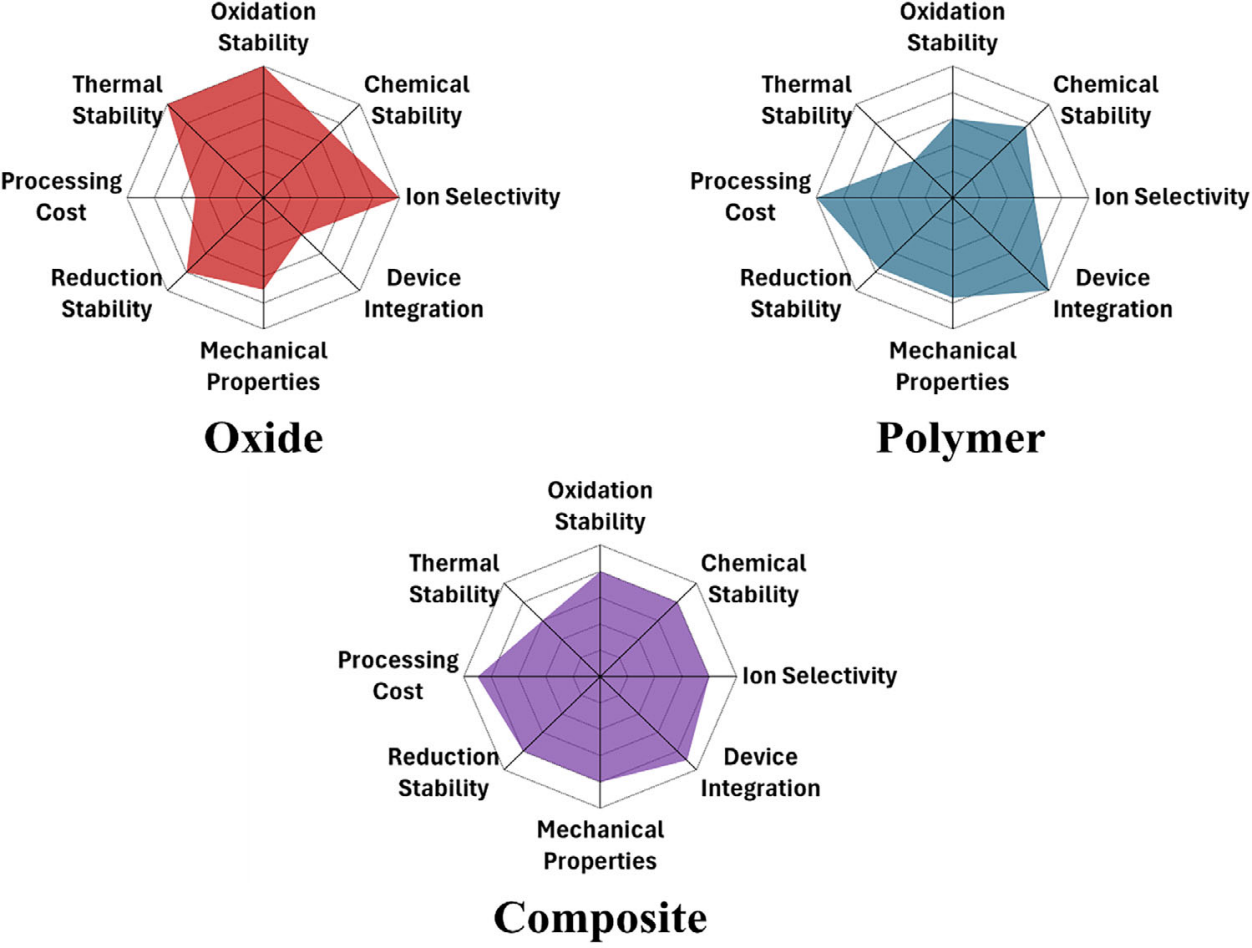
Comparative radar chart of oxide, polymer, and polymer‐oxide composite SEs.^[^ ^6^, ^15^, ^19^ ^]^ Oxide and polymer radar charts. Reproduced with permission.^[^ ^6^ ^]^ Copyright 2025, Elsevier Ltd.

Realistically, no SE can achieve optimum performance in all four categories and compromises are made depending on the material system selected. Discussed in the following sections are the advantages and disadvantages of oxide, polymer, and composite electrolytes. The discussion is summarized in **Table** **1** and **Figure** **1**.

**Table 1:** Advantages and disadvantages of different types of SEs. Asterisks (*) indicate qualitative processibility of the SEs. A larger number of asterisks corresponds to higher processibility.

SE material	Advantages	Disadvantages		Processibility
Polymer	Mechanically flexible Easy process integration		Low conductivity at room temperature Low electrochemical stability window	*****
Oxide	High conductivity Dendrite formation prevention		High‐temperature synthesis Fragile Difficult to process	**
Composite	Mechanically flexible Improved conductivity		Low electrochemical stability window Increased processing complexity depending on filler type	****

Oxide SEs, such as garnet‐type Li_7_La_3_Zr_2_O_12_ (LLZO) or perovskite‐type LiLaTiO_3_ (LLTO), are attractive due to their relatively high ionic conductivity, which can reach up to 10^−3^ S cm^−1^.^[^ ^8^ ^]^ Furthermore, their wide electrochemical stability window allows for greater compatibility with common cathode materials and lithium‐metal anode.^[^ ^9^, ^10^ ^]^ However, the perovskite‐type LLTO is more susceptible to titanium reduction when in contact with lithium metal which can reduce performance.^[^ ^11^ ^]^ Despite their large electrochemical window and relatively high ionic conductivity, the oxide type SEs suffer from high rigidity which can lead to a loss of contact during volume changes during cycling.^[^ ^6^, ^11^ ^]^

Polymer SEs are able to overcome the interfacial contact issues inherent in oxide SEs due to their pliability.^[^ ^12^ ^]^ Furthermore, with polymer binder presence within the composite cathode, a more seamless cathode/electrolyte interface can be generated. The polymer is made conductive by adding lithium salt which complexes with various functional groups. This allows lithium diffusion via ion hopping or chain motion.^[^ ^13^ ^]^ However, polymer SEs have low room temperature ionic conductivity (10^−8^–10^−6^ S cm^−1^) along with a generally narrower electrochemical stability window which can limit the selection of suitable cathode materials.^[^ ^14^, ^15^ ^]^ Methods to overcome the low inherent ionic conductivity include adding plasticizer to promote amorphous regions (main ion‐conducting pathways) or adding crosslinkers to also enhance amorphous regions without sacrificing mechanical stability.^[^ ^13^, ^16^ ^]^ Another route includes single ion‐conducting polymer electrolytes which possess similar ionic conductivities as compared to other polymer SEs. However, they exhibit high lithium transference numbers which enable effective material utilization.^[^ ^17^ ^]^

To overcome the interfacial shortcomings of oxide SE and poor ionic conductivity of polymer SE, composite electrolytes combining polymers with oxide fillers have been developed. Incorporating inert oxide materials can reduce the crystallinity of the polymer matrix which in turn improves the ionic conductivity. Moreover, the oxide filler actively interacts with lithium ions, resulting in ionic conductivity increase. In these composite structures, the polymer remains in contact with the electrodes, ensuring good interfacial contact during cycling while the oxide on its own cannot.^[^ ^18^ ^]^

#### 2.1.2 Cathode Active Materials for Solid‐State Batteries

The cathode active material (CAM) is key to the performance of SSBs acting as the host for intercalated Li‐ions during discharge and Li‐ion source during charging.^[^ ^20^ ^]^ To successfully serve these functions during cell operation, there are several desired characteristics to consider. A high specific capacity allows more charge storage per unit mass increasing the overall capacity of the battery, a high operating voltage to generate a large electrochemical driving force, and fast lithium and electron diffusion to support a higher charge/discharge rate.^[^ ^21^ ^]^ In addition to intrinsic electrochemical performance properties, electrochemical stability against detrimental CAM/SE side reactions and mechanical stability against cycling induced volume changes are critical.^[^ ^22^ ^]^ These stability considerations are even more important for SSBs as the lack of pore‐filling liquid electrolyte requires the use of a composite cathode (i.e., CAM and SE particle mixtures) to improve ionic conductivity within the cathode layer itself as discussed in Section 2.2. Therefore, mechanical stability to maintain contact between CAM and SE during cycling for efficient lithium transport and the electrochemical stability of the CAM to accommodate varied electrochemical windows for different SEs must be given additional consideration when discussing the merits of different CAMs used in SSBs.^[^ ^23^ ^]^

One of the first CAMs used in LIBs was LiCoO_2_ (LCO) which continues to see wide use in electronics due to its relatively high energy density, but it generally possesses a shorter cycle life as compared to other CAMs. Furthermore, LCO exhibits a somewhat low practical capacity compared to the theoretical value (160 vs 274 mAh g^−1^).^[^ ^24^ ^]^ Despite these limitations, high material density along with high lithium and electron conductivity are beneficial to SSBs which suffer from inherently high transport resistances.^[^ ^25^ ^]^ Additionally, increasing the cut‐off potential can mitigate the low capacity of LCO. However, this could cause detrimental phase transitions within the LCO crystal structure causing cracking and an increase in parasitic side reactions.^[^ ^26^ ^]^ Application of LCO with chemically stable SEs with high ionic conductivity, along with strategies to increase the stability of LCO itself (such as surface coatings or doping) can make use of the benefits provided by the denser LCO particles despite the lower capacity.^[^ ^25^ ^]^ To improve the capacity of LCO‐type materials, the incorporation of nickel and additional stabilizing metals (such as manganese or aluminum) into the LCO structure has been widely explored.

LiNi*_x_* Co*_y_* Mn_1−_ *_x_*_−_ *_y_* O_2_ (NCM) has been well used in LIBs as an effective CAM with relatively high capacity. This capacity increases with increasing nickel (Ni) content; however, capacity retention is diminished, indicating reduced stability. Additionally, cycling‐induced volumetric changes within NCM during lithium intercalation lead to cracking and internal pore formation which increases internal diffusion resistance. Surface coating of NCM particles can reduce reactivity with electrolyte while synthesis of single crystal NCM can reduce the microcracking improving the applicability of NCM in SSBs.^[^ ^26^, ^27^ ^]^ LiNi*_x_* Co*_y_* Al_1−_ *_x_*_−_ *_y_* O_2_ (NCA) has a similarly high capacity to Ni‐rich NCM, making it an attractive material to pair with lithium metal within SSBs. However, it suffers from similar degradation mechanisms as NCM.^[^ ^27^, ^28^ ^]^ To create an effective cathode layer using NCM or NCA, an electrolyte capable of accommodating large volume changes would be ideal. The higher operating voltages must also be accounted for requiring an SE that is stable across a larger voltage window.

LiNi_0.5_Mn_1.5_O_4_ (LNMO) has a lower theoretical capacity compared to NCM or NCA, but its very high operating voltage results in a relatively high energy density. Furthermore, the absence of cobalt, which is a critical material, reduces the cost and environmental impact as compared to previously discussed CAMs. At the high operating potential of LMNO, further emphasis must be placed on reducing the detrimental side reactions with the electrolyte during cycling and the oxidation state changes of manganese.^[^ ^29^, ^30^ ^]^ Also, the high degree of volume change during cycling requires a mechanically robust electrolyte.^[^ ^31^ ^]^ It is difficult to obtain an SE that meets both requirements, therefore, a greater emphasis on microstructure/surface engineering of LMNO particles may be more productive.^[^ ^30^, ^32^ ^]^

LiFePO_4_ (LFP) is another commonly utilized CAM that benefits from utilizing noncritical materials. Furthermore, as compared to other lithium‐metal oxides, LFP is very stable during cycling with minimal volume change.^[^ ^33^ ^]^ The operating voltage is also relatively low reducing the need for exceptionally high stability SEs.^[^ ^22^ ^]^ Though LFP is highly stable, the capacity is lower compared to Ni‐rich chemistries and suffers from low electronic conductivity. This encourages the use of surface coating, such as carbon, to improve performance which may limit SE selection to those compatible with the carbon surface for example.^[^ ^22^, ^33^ ^]^

#### 2.1.3 Interfacial Considerations in Selecting CAM–SE Pairs

While the intrinsic properties of the CAM are important in SSBs, the compatibility between the CAM and the selected SE—including factors such as electrochemical stability within the voltage window, chemical stability, and mechanical stability—is of primary importance. This general compatibility is summarized in **Table** **2**.

**Table 2:** Compatibility of CAMs with discussed SEs.

CAM	SE compatibility
LCO	Oxide, composite
NMC	Composite
NCA	Composite
LMNO	Oxide
LFP	Polymer, composite

Electrochemical compatibility between polymer SE and CAM is a critical design constraint in SSBs. When the cathode is charged to a potential above the polymer SE's anodic stability limit, the electrolyte undergoes oxidative decomposition; electrons are withdrawn from states near the electrolyte's highest occupied molecular orbital (HOMO), producing parasitic reactions and resistive interphases that increase interfacial impedance and degrade cycle life. Due to its compatibility with lithium salts and ease of processing, poly(ethylene oxide) (PEO) is the most widely utilized for polymer‐based SEs.^[^ ^34^ ^]^ However, PEO‐based polymer SEs are typically unstable above about 4.0 V versus Li/Li^+^, which is why LFP—whose operating voltage is lower—is commonly paired with polymer SE.^[^ ^35^, ^36^ ^]^

To enable polymer SEs with higher‐voltage cathodes such as NCM and LMNO, the anodic electrochemical window must be widened. A practical route is to lower the HOMO energy by introducing high‐polarity, electron‐withdrawing functional groups (such as sulfone or cyano) into the polymer backbone or side chains, thereby improving oxidative stability.^[^ ^37^ ^]^ These molecular design strategies can be combined with appropriate salts and composite architectures to balance enhanced stability with sufficient ionic conductivity and mechanical integrity. Alternatively, methods such as crosslinking or copolymerization can be employed to design polymers with enhanced antioxidative properties.^[^ ^38^, ^39^ ^]^ For example, copolymerized PEO‐tetraglyme electrolytes demonstrate an excellent electrochemical stability up to 5.0 V versus Li/Li^+^.^[^ ^40^ ^]^ In addition to modifying the polymer SE itself, coating the surface of cathode active materials to prevent interfacial degradation is also gaining attention. These approaches will be discussed in detail in Section 3.1.

For oxide SEs, achieving intimate contact with CAMs typically requires high‐temperature cosintering (>500 °C). Under these conditions, interfacial interdiffusion and solid‐state reactions can occur, so chemical compatibility must be carefully considered in materials selection. These reactions frequently generate impurity phases at the interface, which lower Li‐ion conductivity and increase interfacial resistance.^[^ ^41^, ^42^, ^43^ ^]^ For instance, while LCO is thermally stable and thus compatible with LLZO, numerous secondary phases, including LaCoO_3_ and La_2_Zr_2_O_7_, have been reported as a function of processing conditions.^[^ ^43^, ^44^ ^]^ Consequently, judicious pairing of SE and CAM compositions—potentially with protective cathode coatings and controlled sintering profiles—is essential to preserve interfacial transport and cell performance. To prevent this, approaches such as introducing a buffer layer and using sintering additives are being adopted, and these will be discussed in Sections 3.1 and 3.3.

Composite cathode performance is governed by the co‐design and optimization of the SE, CAM, and their compatibility. Beyond intrinsic material properties, practical performance is dominated by voltage window alignment, chemical reactivity, mechanical contact, and processing conditions. Polymer SEs are best paired with lower‐voltage CAMs or must have their anodic stability window widened via molecular design. Oxide SEs, which often require high‐temperature cosintering, demand mitigation of secondary phases and interfacial reactions through interlayers and low‐temperature densification. The selection principles in Section 2.1 are applied in concert with microstructure‐engineering strategies to enable balanced ionic/electronic transport and durable cycling in thick, high‐loading composite cathodes.

### 2.2 Cell Structure and Challenges in Cathode Microstructure

**Figure 2:**
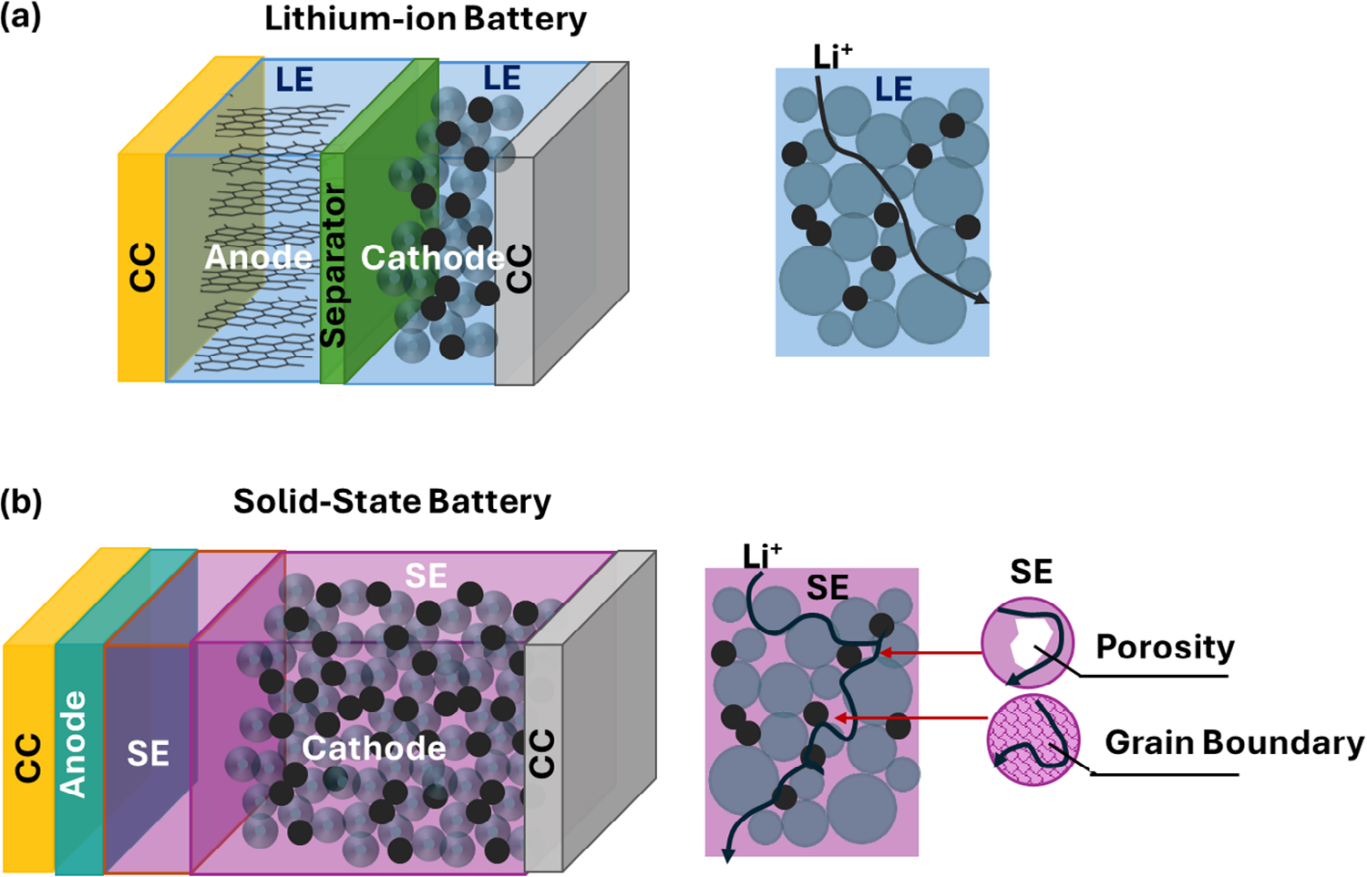
Schematic illustrations of cell structures and lithium‐ion transport pathways in a) an LIB cell and b) an SSB cell.^[^ ^22^ ^]^

The primary difference between SSBs and traditional LIBs lies in the type of electrolyte used; SSBs employ SEs instead of the LEs deployed in LIBs, as illustrated in **Figure** **2**. Unlike LEs, SEs cannot infiltrate the voids and gaps within the cathode structure. As a result, SSB cathodes typically require a composite structure in which SE materials are directly integrated into the cathode.

**Figure 3:**
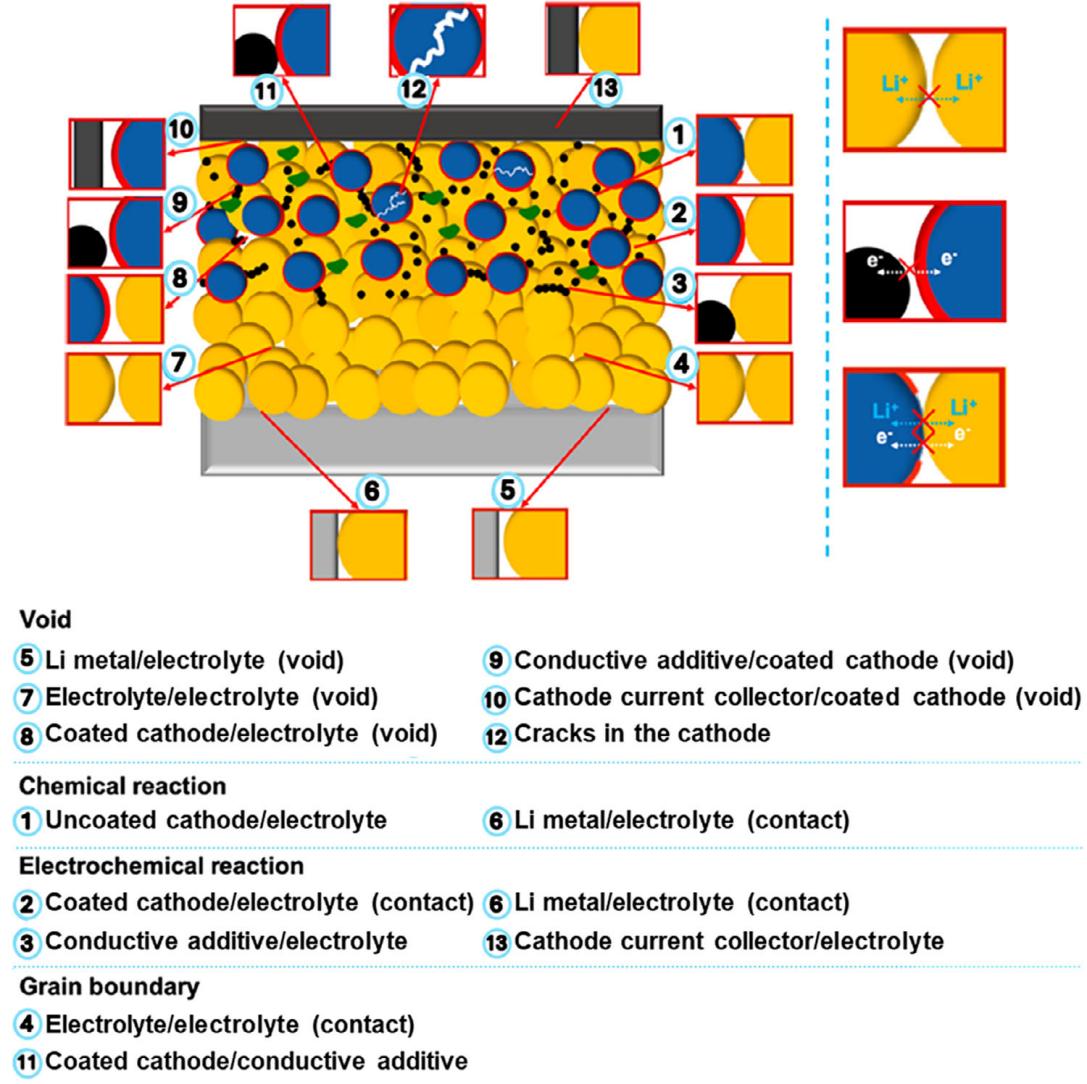
Schematic illustration of interfacial phenomena in SSBs. Reproduced with permission.^[^ ^47^ ^]^ Copyright 2020, American Chemical Society.

The unique electrochemical, transport, and mechanical properties of SEs, as well as the nature of the solid/solid interfaces between SEs and the active materials, introduce new challenges in the design and synthesis of SSB cathodes. On one hand, maximizing the volume fraction of CAMs is crucial for achieving high energy density. On the other hand, maintaining sufficient fractions of SEs and electronically conductive additives, such as carbon black, is necessary to ensure adequate ionic and electrical conductivity, which is vital for achieving high power density. Therefore, a carefully balanced cathode composition is essential. In addition to composition, the spatial arrangement of CAMs, SEs, and electronically conductive additives must be optimized to enable effective ion and electron transport. This is especially important in SSBs, where the tortuosity of ionic pathways is significantly increased (**Figure** **3**).^[^ ^22^, ^45^ ^]^ Experimental studies have demonstrated that a CAM volume fraction of 50 vol% is achievable,^[^ ^46^ ^]^ while 70% is expected to achieve optimized cathode structures.^[^ ^47^ ^]^

Beyond transport‐related challenges, interfacial instability between CAM and electrolyte represents another critical issue in the development of SSBs. As shown in Figure 3, this instability can arise from interionic diffusion (thermodynamic instability), chemical reactions due to mismatched chemical potentials, or electrochemical reactions such as the oxidation or reduction of SE under high‐ or low‐voltage conditions when in contact with electrically conductive CAM.^[^ ^47^ ^]^

Other complications include high interfacial resistance and overvoltage resulting from porosity in insufficiently densified cathodes,^[^ ^48^ ^]^ limited high current applications and low charging rate, as well as ionic conductivity degradation caused by grain boundaries. Depending on the mechanical properties of the cathode materials and the conditions of their synthesis and calendaring processes, cathode porosity can range from 10% to 40%. Furthermore, cracks formed during cycling may lead to electrode pulverization and the formation of additional voids. Grain boundaries can form due to electrical potential differences between CAMs and conductive additives, which may lead to lithium‐deficient space‐charge layer at the CAM/conductive additive interface after Li‐ion transfer from one to another. Due to the low ionic conductivity of the SE and composite cathode, as well as inhomogeneous Li‐ion diffusion caused by polarization, current SSBs are still unable to operate at high critical current densities (CCDs) or support high charging rates.^[^ ^6^ ^]^

To address these challenges, various mitigation strategies are necessary, such as selecting chemically compatible combinations of CAM and SE, applying protective inert coatings to CAM particles, improving SE material ionic conductivity and stability, developing nanostructured cathode materials, optimizing cathode microstructure, creating advanced cathode architectures, and matching materials based on their operating voltage windows, among other approaches. We will discuss these strategies in the following sections.

### 2.3 Electrochemical Characterization for Solid‐State Batteries

Component‐level testing is essential for evaluating the properties of individual materials such as SEs and active materials, as well as their interfaces. Key metrics in these tests include ionic conductivity (measured in S cm^−1^), electronic conductivity (*σ*_e_, typically very low in electrolytes), the Li⁺ transference number, and the CCD for lithium plating. To provide a clear overview of these metrics and their corresponding measurement methods, we have compiled a table that summarizes the essential component metrics, and the techniques used to assess them (**Table** **3**). Ionic conductivity is often assessed using electrochemical impedance spectroscopy (EIS) on a dense pellet with blocking electrodes. The stack pressure, sweep frequency range, and measurement temperature(s) are essential parameters for comparison between materials. In one study, researchers pressed Li_6_PS_5_Cl argyrodite into a pellet at 380 MPa for 3 min and conducted potentiostatic EIS across a frequency range from 7 MHz to 100 mHz, with a 10 mV amplitude, at temperatures between 10 and 60 °C under a stack pressure of 60 MPa.^[^ ^49^ ^]^ Electronic conductivity is typically determined through Hebb–Wagner or DC polarization methods in mixed (ion blocking) cells. CCD is measured by galvanostatically ramping the current in a Li|SE|Li symmetric cell (or a Li‐free half‐cell) until a short circuit occurs.^[^ ^50^, ^51^ ^]^ Other important component‐level metrics include the electrochemical stability window, evaluated via cyclic voltammetry or linear sweep voltammetry, and mechanical properties including elastic modulus, hardness, and fracture toughness measured by indentation or compression tests. Interfacial stability is inferred from the area‐specific interface resistance (Ω cm^2^), measured by EIS of electrode|SE interfaces or through long‐term plating tests. Component testing is usually conducted under controlled temperatures, commonly ≈25 °C, with some tests sweeping from 10 to 75 °C for Arrhenius analysis. Additionally, applied pressure ranging from tens to hundreds of MPa is often used to ensure solid contact.^[^ ^49^ ^]^

**Table 3:** Solid‐state battery testing: component metrics and methods.^[^ ^49^, ^50^, ^51^, ^52^ ^]^

Component‐level parameter	Measurement method	Typical conditions/notes
Ionic conductivity (*σ*) [S cm^−1^]	Potentiostatic EIS on dense pellet with blocking electrodes	Pellet pressed (≈100–400 MPa) to 2000 µm; measured under stack pressure (≈50–100 MPa) at 10–60 °C, Ar atmosphere
Electronic conductivity (*σ*_e_) [S cm^−1^]	DC polarization (Hebb–Wagner) on mixed‐conducting cell	Same pellet, Li‐blocking electrodes; very low values
Li⁺ transference number	DC polarization (Bruce–Vincent method) + EIS	Typically, at 25 °C on symmetric cell
Critical current density (CCD) [mA cm^−^^2^]	Galvanostatic current ramp in Li	SE
Mechanical properties (*E*, hardness)	Nanoindentation, compression test (pellet)	On bulk samples, indicates fracture resistance
Stability window (V vs Li)	Cyclic voltammetry or linear sweep voltammetry (Li vs inert counter)	Typically, 0–5 V to find oxidation/reduction limits
Interface resistance [Ω cm^2^]	EIS of electrode	SE interface (symmetric or composite cell)

Full‐cell tests are crucial for evaluating the electrochemical performance of assembled batteries (cathode|SE|anode). Key metrics in these tests include “specific capacity” (mAh per gram of active material or per cm^2^), “Coulombic efficiency” (%), “cycle life” or “capacity retention,” and “rate capability.” These parameters are typically measured by galvanostatic charge–discharge cycling, either in constant current (CC) mode or constant current–constant voltage (CC‐CV) mode. For instance, one study demonstrated that an NCM–sulfide SSB cycled at 0.5 C for 250 cycles exhibited ≈99.2% capacity retention.^[^ ^50^ ^]^ Another study reported a SnCl_2_‐doped Li_2_P_2_S_8_I‐based SSB with an initial discharge capacity of 183 mAh g^−1^ at 0.1 C and a first‐cycle efficiency of about 74%, which improved to ≈98% retention after 100 cycles.^[^ ^50^ ^]^ Coulombic efficiency, calculated as the ratio of discharge to charge capacity (*Q*_discharge/*Q*_charge), often exceeds 98–99% at steady state. “Rate performance” is assessed by cycling at various *C* rates, such as 0.1, 0.5, and 1 C, and comparing the resulting capacities. EIS in full cells is used to monitor total cell resistance, measured in Ω cm^2^, before and after cycling, typically employing a small‐amplitude EIS at open‐circuit voltage (OCV) across the range from 1 MHz to 0.01 Hz). Cells are generally assembled using a pressed‐stack fixture, with stack pressures ranging from 5 to 400 MPa.^[^ ^49^ ^]^ Most studies reported using pressures between 10 and 50 MPa to ensure cycling stability. Cycling performance is typically evaluated at ≈25 °C, although some experiments are performed at higher temperatures, such as 60 °C, to improve kinetics, or at lower temperatures, down to –20 °C), to evaluate cold performance. **Table** **4** presents a concise summary of these metrics, and the test methods used to measure full cells.

**Table 4:** Solid‐state battery testing: full‐cell metrics and methods.^[^ ^49^, ^50^, ^52^, ^53^, ^54^ ^]^

Full‐cell metric	Test method	Typical conditions/notes
Specific capacity [mAh g^−1^ or mAh cm^−^^2^]	Galvanostatic charge/discharge cycling (CC mode, CC‐CV mode)	For example, 2.5–4.3 V cutoff, rates 0.05–1 C; report per active mass or area; e.g., 180 mAh g^−1^@0.1 C
Coulombic efficiency [%]	From cycling data (*Q*_discharge/*Q*_charge)	First‐cycle CE may be <80% (SEI formation); typically >98% after formation
Cycle life/capacity retention	Long‐term cycling (100–1000+ cycles)	Report % capacity after *N* cycles (e.g., 99.2% after 250 cycles at 0.5 C)
Rate capability	Cycling at varied *C*‐rates (capacity vs *C*‐rate)	Test at multiple rates (0.1–1 C); measure drop in capacity at high rate
Area‐specific impedance [Ω cm^2^]	EIS of full cell (high freq. intercept and semicircle)	Measured at OCV or after charge; tracks total cell (electrode + interface) resistance
Energy density [Wh kg^−1^]	Calculated from capacity × average voltage	Depends on cell mass; often projected for SSBs

Recent advancements in materials and cell design indicate that SSBs can achieve specific energy exceeding 250 Wh kg^−1^ while maintaining stable long‐term cycling performance.^[^ ^3^, ^55^, ^56^, ^57^ ^]^ However, high specific energy alone is insufficient for practical adoption; it must be complemented by high specific power (>250 W kg^−1^), low internal resistance (<40 Ω cm^2^), and sustained efficiency and durability over thousands of cycles.^[^ ^5^, ^52^, ^58^ ^]^

A well‐designed SSB must integrate multiple parameters. The cell voltage should exceed 4 V, with practical specific capacities approaching theoretical values at room temperature, such as 200 mAh g^−1^ for NCM, 140–150 mAh g^−1^ for LCO and LNMO, and 170 mAh g^−1^ for LFP.^[^ ^52^, ^59^ ^]^ Nonetheless, SSBs utilizing NCM cathodes often underperform, typically yielding ≤150 mAh g^−1^, whereas NCM‐based CAMs can deliver up to 200 mAh g^−1^ in LE systems.^[^ ^60^, ^61^ ^]^ Bridging this performance gap necessitates increasing the CAM volume fraction in composite cathodes, ideally surpassing 70 vol%, and incorporating ultrathin solid electrolyte layers (<30 µm) to minimize ionic path lengths and internal resistance.^[^ ^62^, ^63^ ^]^ Cathode composites aim to achieve a hypothetical specific energy of ≈500 Wh kg^−1^ (vs Li⁺/Li by cathode mass) and an areal capacity of 5 mAh cm^−^^2^.^[^ ^64^, ^65^ ^]^ To support high energy and current densities without dendrite formation, SEs must exhibit ionic conductivities greater than 10 mS cm^−1^ and robust electrochemical stability, while delivering energy efficiencies exceeding 90%.^[^ ^52^, ^66^ ^]^ Achieving these metrics requires SEs with high ionic conductivity, minimal electrochemical degradation, and reduced thickness to lower transport resistance and enhance volumetric energy density. Similarly, the thickness of cathode composites must balance increased energy with manageable resistance, remaining within limits necessary to support high‐rate capability. Achieving system‐level performance targets requires innovations in manufacturing processes. A key objective is the scalable fabrication of large‐area pouch cells (>200 cm^2^), while maintaining energy efficiencies above 90% and ensuring long‐term durability, with energy retention exceeding 80% over 1000 cycles and delivering at least 5 Wh cm^−^^2^ of area‐specific energy.^[^ ^49^, ^52^ ^]^ The industry trend also focuses on reducing lithium‐metal thickness and avoiding excessive external pressure during cycling—conditions critical for practical applications. These target performance metrics are summarized in **Table** **5**.

**Table 5:** Target performance of solid‐state battery.^[^ ^52^, ^60^, ^62^, ^65^, ^66^, ^73^, ^74^, ^75^, ^76^ ^]^

METRICS	TARGET
Cell voltage, V [V]	>4 V
Specific capacity, *q* [mAh g^−1^]	Needs to reach a theoretical value at ambient temperature, e.g., 200 mAh g^−1^ for NCM
Volume fraction of cathode active materials, *Φ*_CAM_	70 vol%
Hypothetical specific energy, *E*_m(Ca)_	250–500 Wh kg^−1^ (vs Li^+^/Li by weight of cathode only)
Area capacity of composite cathode, *Q*_A_	5 mAh m^−2^
Current density, *j*	5 mA cm^−2^
Energy efficiency, *Φ*_E_	>90%
Internal resistance, *R*	<40 Ω cm^−2^
Solid electrolyte thickness, *I*_SE_	As little as 30 µm has been reported^[^ ^62^, ^63^ ^]^
Cathode thickness, *I*_Ca_	A balance of increasing specific energy of the cell by increasing thickness, while remaining within a practical window for the required current density
Cell area, *A*	Pouch cells require >200 cm^2^
Energy retention	>80% over 1000 cycles
Pressure, *p*	Excessive pressure should be avoided

To ensure reproducibility and fair benchmarking, the full test context should be reported (temperature profile, stack pressure, cell geometry, Ω cm^2^ normalization, SE/cathode thickness, and CAM loading/porosity). Prospectively, integrating operando and postmortem characterization with multiphysics models will quantify interfacial resistance growth, tortuosity evolution, and failure signatures, clarify how microstructure and interfaces govern performance, and accelerate optimization toward thick, high‐loading cathodes. The pathway to high‐performance SSBs involves the co‐optimization of material properties, electrode architecture, electrolyte characteristics, and scalable fabrication methods. By aligning with these interconnected targets, SSBs can move closer to commercial readiness for demanding applications such as electric vehicles and grid‐scale energy storage.

While electrochemical testing provides vital insights into the overall performance of SSBs, these macroscopic results are often influenced by complex interfacial phenomena between the SE and electrodes. To fully understand the mechanisms governing charge transfer, interfacial resistance, and long‐term stability, it is essential to complement electrochemical measurements with detailed interface characterization. A suite of advanced tools has therefore been applied to probe interface morphology and chemistry directly in SSBs, such as high‐resolution microscopy techniques (e.g., scanning electron microscopy (SEM) with focused ion beam (FIB) cross‐sectioning, energy‐dispersive X‐ray spectroscopy (EDS), and transmission electron microscopy (TEM)), complementary spectroscopic methods (e.g., X‐ray photoelectron spectroscopy (XPS)), and in situ and operando techniques (e.g., in situ optical/electron microscopy, X‐ray diffraction (XRD), TEM, XPS, and Raman spectroscopy).^[^ ^67^, ^68^, ^69^, ^70^, ^71^, ^72^ ^]^ Such analyses elucidate the chemical composition, structural evolution, and morphological changes occurring at the electrode–electrolyte interfaces, and guide electrode microstructure and interface engineering (e.g., protective coating or buffer layers) to mitigate degradation. Building on these insights, recent efforts have increasingly focused on interface microstructure engineering, aiming to manipulate interfacial architecture and composition to enhance ionic contact, suppress degradation, and achieve stable, high‐performance SSBs.

## 3 Recent Progress in Cathode Microstructure Engineering for SSBs

### 3.1 Improving SE–CAM Contact through Active Materials’ Coatings

SSBs often experience high interfacial resistance and degradation at the cathode/electrolyte boundary. To address these challenges, coating cathode particles with protective layers has emerged as a pivotal strategy to enhance cycle life and rate performance.^[^ ^77^ ^]^ These cathode coatings act as artificial interphases, similar to cathode electrolyte interphase (CEI) layers, by physically separating the CAM from the SE, inhibiting parasitic reactions, and preserving the structural integrity of the cathode. A diverse array of coating chemistries has been explored, ranging from simple oxides and phosphates to complex hybrid and polymer layers, each tailored to complement the specific cathode chemistry and electrolyte type.

#### 3.1.1 Coating Materials and Functionalities

A broad spectrum of coating materials has been investigated for their compatibility with various cathode chemistries and electrolyte types. Among these, metal oxides, phosphates, halides, polymers, and composite materials have shown promising results.

Inorganic oxides are widely utilized as inert barriers in batteries with both LE and SE. Alumina (Al_2_O_3_), zirconia (ZrO_2_), titania (TiO_2_), and other stable oxides have been traditionally applied for their protective properties.^[^ ^78^, ^79^, ^80^, ^81^ ^]^ More recently, lithium‐containing oxides, such as LiNbO_3_, Li_4_Ti_5_O_12_, and Li_2_ZrO_3_, have gained favor due to their ability to facilitate Li⁺ transport while blocking electron passage.^[^ ^77^ ^]^ For Ni‐rich layered cathodes, LiNbO_3_ is particularly prevalent, as it effectively suppresses cathode surface reactions and limits oxygen release at high voltage.^[^ ^77^ ^]^ Moreover, novel oxides like Li_5_TaO_5_, LiNbO*_x_*, and LiTaO_3_ are being studied for their chemical stability and ionic conductivity, offering potential advancements in cathode coating technology.^[^ ^77^ ^]^

Building upon the role of metal oxides, lithium phosphates and borates have emerged as alternative protective layers that form dense and stable coatings resistant to oxidation. These materials, such as Li_3_PO_4_ or LiTi_2_(PO_4_)_3_, form dense and stable coatings that resist oxidation and prevent the decomposition of sulfide electrolytes. For example, thin layers of Li_3_PO_4_ or LiTi_2_(PO_4_)_3_ applied to NCM cathodes have been shown to suppress SE decomposition and significantly enhance cycling stability.^[^ ^82^ ^]^ Similarly, lithium borate materials such as Li_3_BO_3_ have been proposed as low‐reactivity interlayers, providing additional protection against degradation.^[^ ^77^ ^]^

Expanding on these materials, lithium halides such as LiF, LiI, and Li_3_InCl_6_ are emerging as promising coating candidates, owing to their high electrochemical stability and compatibility with solid electrolytes. For instance, LiI has been added into oxide coatings to boost Li⁺ conductivity. A composite coating of LiInO_2_–LiI on Ni‐rich NCA cathodes formed an in situ LiI‐derived interphase with thiophosphate electrolytes, which greatly improved rate capability and interface conductivity.^[^ ^83^ ^]^ Similarly, nanocrystalline halide electrolytes, such as Li_3_InCl_6_, have been used as cathode shell coatings to stabilize sulfide interfaces. These halide layers effectively limit interfacial reactions while providing fast Li⁺ pathways, contributing to improved overall battery performance.

In parallel with inorganic options, polymers and hybrid organic–inorganic materials have garnered attention as flexible and adaptable interlayers. Conducting and insulating polymers have been increasingly explored as flexible interlayers.^[^ ^78^ ^]^ Conductive polymers, such as poly(3,4‐ethylenedioxythiophene) (PEDOT), polyaniline (PANI), poly(3,4‐ethylenedioxythiophene) polystyrene (PS) sulfonate (PEDOT:PSS), and polyacrylate, can form amorphous, conformal films that not only block electron passage but also facilitate Li⁺ transport. For example, ball‐milled PANI coatings on LCO serve as elastic buffer against a sulfide electrolyte, significantly reducing interfacial resistance and enabling 85.5% capacity retention over 200 cycles in Li─PS─Cl SSB cells.^[^ ^84^ ^]^ Similarly, ultrathin PEDOT:PSS films (≈5–20 nm) applied to Ni‐rich layered oxides have been shown to enhance both Li⁺ and electron conduction, suppress phase changes, and prevent cracking during cycling.^[^ ^78^ ^]^ Polymers offer advantages over rigid coatings by accommodating strain and mitigating interparticle contact loss, and they can be applied using low‐temperature solution methods or in situ polymerization techniques.

To further optimize performance, advanced coating strategies increasingly employ composites that combine multiple materials. One such approach is the “double‐layer” coating, where each layer serves a specific role—typically, a sulfide‐based film is positioned adjacent to the solid electrolyte for high Li‐ion conductance, while an oxide or halide layer interfaces with the cathode for chemical protection.^[^ ^85^ ^]^ Computational studies have identified material pairs, such as Li_5_B_7_S_13_ (sulfide) + Li_3_YCl_6_ (halide), as outperforming single‐layer coatings.^[^ ^85^ ^]^ Another example is the LiInO_2_–LiI composite,^[^ ^83^ ^]^ which combines a stable oxide with a lithium halide to improve interfacial conductivity. These coatings aim to harness the synergistic benefits of their individual components, delivering both chemical stability and efficient ion transport within a unified coating system.

#### 3.1.2 Mechanisms of Performance Improvement

**Figure 4:**
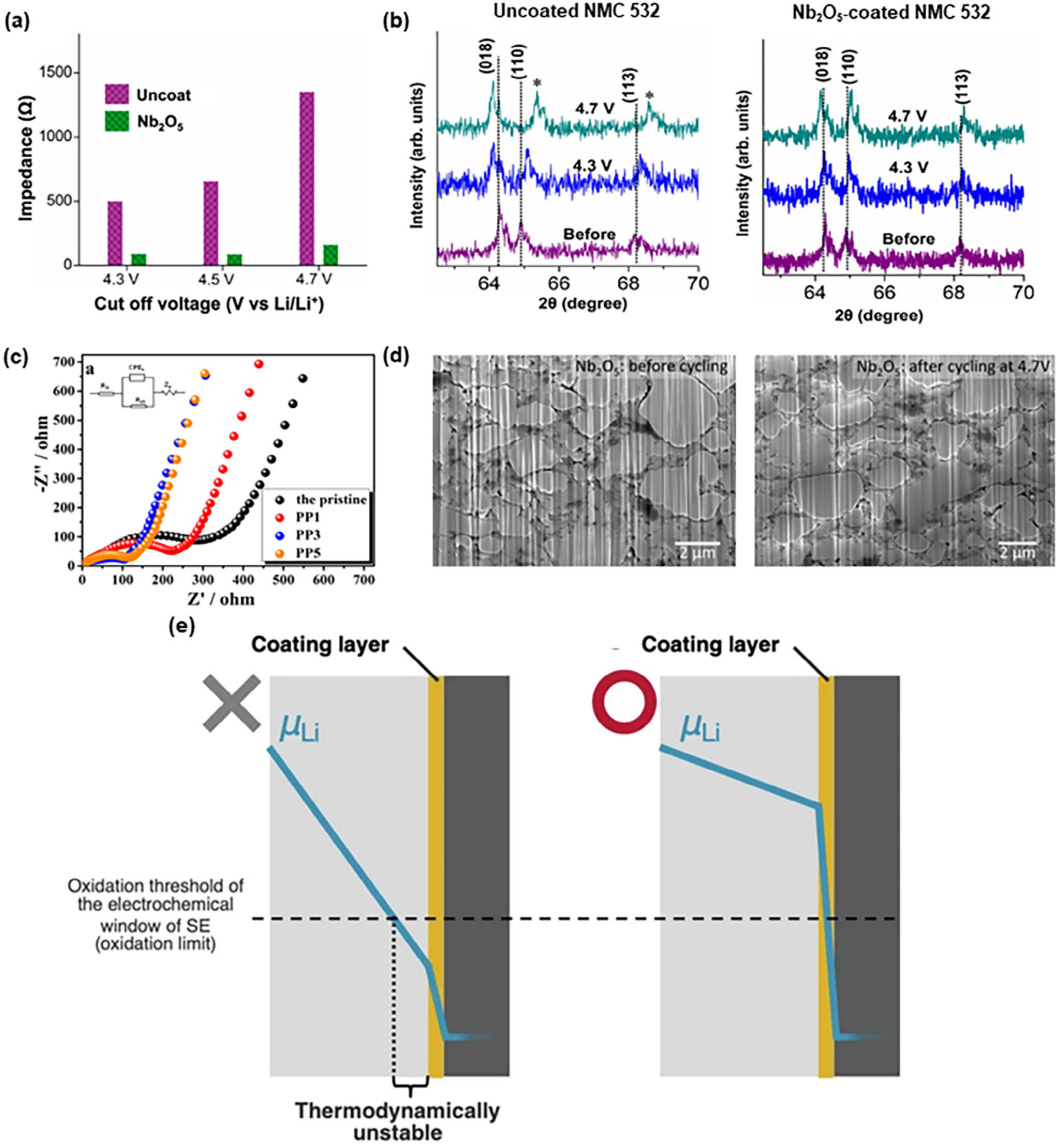
a) Interfacial impedance of uncoated and coated cathodes. b) Ex situ XRD results of uncoated and coated samples after rate performance test. Reproduced with permission under the terms of the Creative Commons CC BY license 4.0.^[^ ^77^ ^]^ Copyright 2024, the Authors. Published by Springer Nature. c) Nyquist plots of the PEDOT:PSS‐coated Li_1.2_Ni_0.2_Mn_0.6_O_2_. Reproduced with permission.^[^ ^86^, ^87^ ^]^ Copyright 2016, American Chemical Society. d) Cross‐sectional scanning electron microscopy (SEM) images of Nb_2_O_5_‐coated NCM before and after cycling. Reproduced with permission under the terms of the Creative Commons CC BY license 4.0.^[^ ^77^ ^]^ Copyright 2024, the Authors. Published by Springer Nature. e) Schematic of the lithium chemical potential (*µ*_Li_) distribution with and without a coating layer. Reproduced with permission under the terms of the Creative Commons CC BY license 4.0.^[^ ^88^ ^]^ Copyright 2024, the Authors. Published by Springer Nature.

Coatings play a crucial role in mitigating interface degradation and boosting performance in SSBs through a variety of mechanisms (**Table** **6**). The primary function of coatings is to prevent direct contact between the high‐voltage cathode and electrolyte. By acting as a barrier, coatings inhibit redox or chemical reactions (e.g., sulfide oxidation to SO*_x_*) that would otherwise lead to the formation of resistive interphases.^[^ ^77^, ^82^ ^]^ This preservation of the cathode surface structure helps limit capacity loss. For example, a HfO_2_ layer on NCM cathodes via atomic layer deposition (ALD) effectively sealed the interface with a lithium thiophosphate SE, resulting in more stable cycling and higher initial Coulombic efficiency.^[^ ^57^ ^]^ Likewise, LiNbO_3_ coatings on Ni‐rich cathodes have been found to “suppress CEI formation” and mechanically block oxygen release at high voltages (**Figure** **4**a,b).^[^ ^77^ ^]^ By preventing direct chemical interaction between the cathode and SE, coatings reduce decomposition reactions, thereby lowering interfacial impedance and extending cycle life.

**Table 6:** Surface coating of active materials.

Coating material	Coating method	Cathode‐active material/electrolyte	Mechanism	Performance	Refs.
ZrO_2_	Sol–gel method	LiNi_0_._90_Mn_0_._05_Co_0_._05_O_2_ (NCM90)	Suppresses interfacial resistance increase and improves structural stability	Discharge capacities of 198.2, 166.1, 156.6, and 75.2 mAh g^−1^ at 0.2, 1, 2, and 10 C at room temperature	[79]
LiNbO_3_	Sol–gel coating	LiNi_0.8_Co_0.1_Mn_0.1_O_2_ (NCM811) LiNi_0.7_Co_0.1_Mn_0.2_O_2_ (NCM712) LiNi_0_._5_Co_0_._2_Mn_0_._3_O_2_ (NCM523) LiCoO_2_ (LCO)	Suppresses cathode surface reactions and release oxygen at high voltages	Capacity retention of 84% after 75 cycles at 0.2 C	[89, 90, 91, 92]
Li_2_ZrO_3_	Sol–gel coating	LiNi_0.6_Co_0.2_Mn_0.2_O_2_ (NCM 622) LiNi_0_._5_Co_0_._2_Mn_0_._3_O_2_ (NCM523)	Acts as a protective layer but may degrade to ZrO_2_, leading to loss of protection	Initial improvement, but degradation observed over 50 cycles	[93, 94]
Li_3_B_11_O_18_	Sol–gel coating	LiNi_0_._5_Co_0_._2_Mn_0_._3_O_2_ (NCM523)	Provides chemical stability and suppresses interfacial reactions	Superior performance compared to Li_2_ZrO_3_‐coated cathodes	[93]
Li_3_PO_4_	Solution‐based coating	LiNi_0_._75_Co_0_._1_Mn_0_._15_O_2_	Acts as a buffer layer to suppress interfacial reactions with sulfide electrolytes	Improved electrochemical performance in all‐solid‐state cells	[95]
Alucone	ALD	LiNi_0.8_Co_0.1_Mn_0.1_O_2_ (NCM811)	Provides a uniform, thin protective layer enhancing interfacial stability	Improved cycling stability and rate performance	[96]
Li_3_PS_4_	Dry coating with annealing	LiNi_0.85_Co_0.10_Mn_0.05_O_2_ (NCM85)	Enhances interfacial contact and stability through tailored annealing	Improved electrochemical performance	[97]
LiPOF	Solution‐based coating	LiNi_0.85_Co_0.10_Mn_0.05_O_2_ (NCM85)	Stabilizes the cathode–electrolyte interface, reducing degradation	Capacity retention of 90% after 100 cycles at 0.2 C	[98]
sPPSLi/PVP	Solution‐based coating	LiNi_0.9_Mn_0.05_Co_0.05_O_2_ (NCM95)	Forms a lithium‐containing polymer layer that enhances ionic conductivity and interfacial stability	Improved specific capacity, especially at high *C*‐rates	[99]

In addition to their role in chemical stabilization, coatings also significantly reduce interfacial impedance, enhance charge transfer, provide pathways for Li‐ion transport, and enable higher‐rate performance. Thin, uniform coatings, often applied using ALD or vapor methods, minimize the additional resistance while effectively protecting the interface.^[^ ^77^ ^]^ An ideal coating is electronically insulating but ionically conductive, with band edges aligned to prevent charge leakage and a minimal thickness to optimize performance.^[^ ^77^ ^]^ For instance, PEDOT or PEDOT:PSS coatings on layered cathodes provide mixed ionic/electronic pathways that accelerate Li⁺ transport at the interface (Figure 4c).^[^ ^78^, ^86^, ^87^ ^]^ In practice, cells with coated cathodes consistently demonstrate higher *C*‐rate capabilities; for example, Ni‐rich NCM–Li_6_PS_5_Cl cells with ALD‐applied HfO_2_ coatings maintain capacity at higher currents than uncoated cells.^[^ ^57^ ^]^

Beyond electrochemical benefits, coatings also function as mechanical buffers. Solid/solid interfaces in SSBs experience significant stress during lithiation and delithiation processes. Coatings can act as elastic interlayers that accommodate volume changes and maintain particle contact at these interfaces, thereby reducing cracking and degradation. Polymer coatings are especially effective in this regard; for example, the flexible PANI film on LCO has been shown to lower and stabilize the LCO/SE interface resistance through cycling.^[^ ^84^ ^]^ Likewise, highly conformal coatings (e.g., few nm ALD films) effectively avoid pinholes or cracks that could concentrate strain, as shown in Figure 4d.^[^ ^77^, ^78^ ^]^ By suppressing microcracks and phase transformations, these coatings contribute to maintaining long‐term capacity retention and high Coulombic efficiency.^[^ ^78^ ^]^

Recent theoretical insights suggest that coatings help regulate the lithium chemical potential at the interface, thereby maintaining the electrochemical stability window of both the cathode and SE (Figure 4e).^[^ ^88^ ^]^ In essence, an effective coating “pins” the lithium potential at the interface, ensuring that neither the cathode nor the SE is driven outside its electrochemical stability range. This thermodynamic protection prevents electrolyte decomposition and enables the SE to remain inert under high‐voltage cathode conditions.^[^ ^88^ ^]^

The field is moving toward increasingly tailored coatings, designed at the nanoscale, to simultaneously address chemical, mechanical, and transport challenges. By combining deposition techniques, such as ALD, wet chemistry and sputtering, with innovative materials like oxides, halides, polymers, and composites, researchers continue to improve SSB performance. These coatings have already enabled many SSBs to achieve hundreds or even thousands of cycles at high rates, underscoring their significance for the next generation of batteries. Despite their benefits, coatings introduce tradeoffs. Overly thick or poor Li^+^‐conductive layers raise charge‐transfer resistance and limit high‐rate operation, brittle oxides can crack under stack pressure, and chemical incompatibilities may yield secondary phases. Therefore, coating chemistry, thickness, and conformality must be co‐optimized and rigorously validated for the specific CAM–SE pair using EIS, XPS, and TEM.

### 3.2 Improve Charge Transportation through Cathode Composition Optimization

Composite cathodes play an important role in SSBs, influencing energy and power densities through their microstructural properties. A typical composite cathode comprises CAM, SE, binder, and conductive additives, collectively facilitating conduction pathways for Li‐ions and electrons.^[^ ^22^ ^]^ Effective microstructure optimization aims at balancing ionic and electronic transport pathways, minimizing tortuosity, and maximizing both capacity and power density.^[^ ^22^, ^100^, ^101^, ^102^, ^103^ ^]^

**Figure 5:**
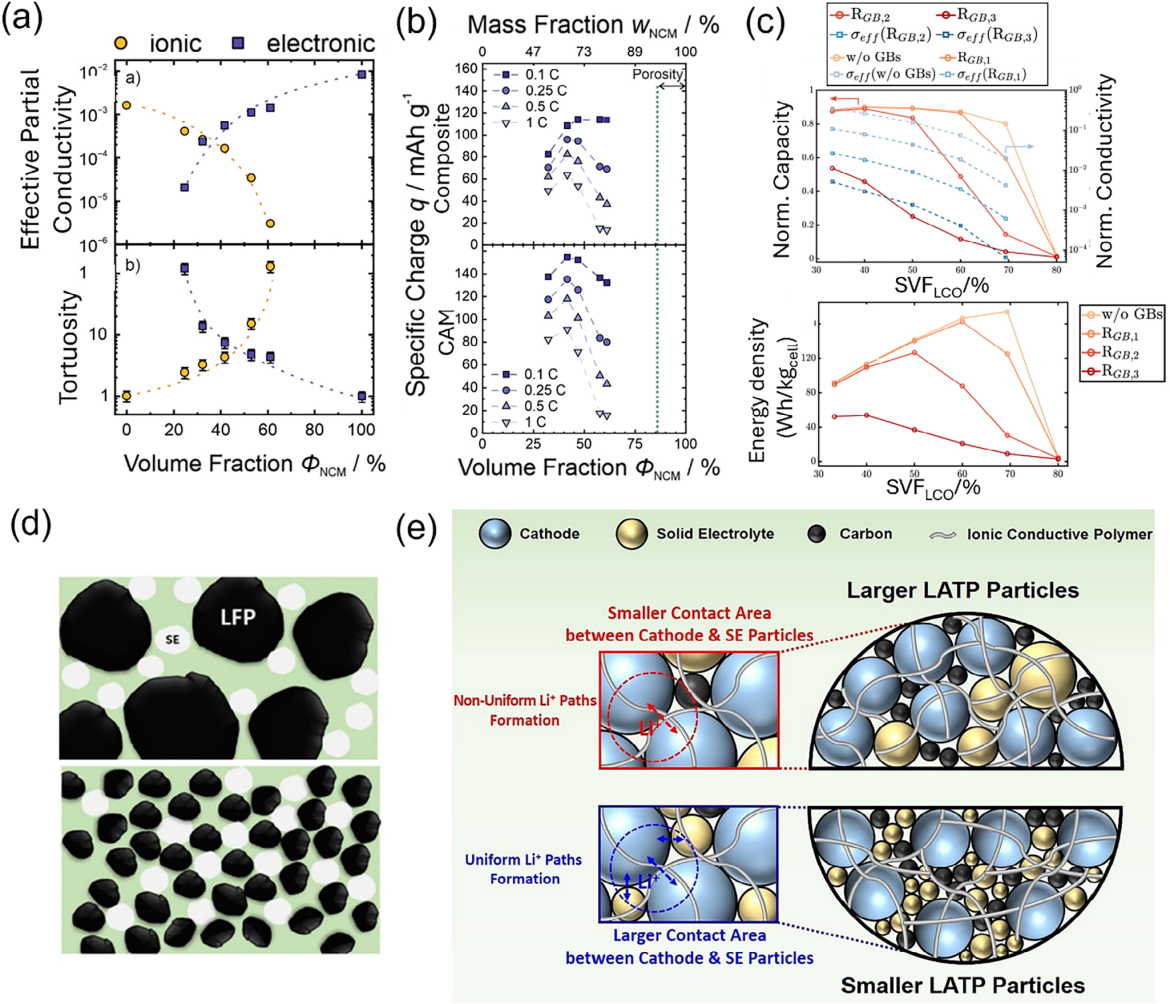
a) Top: Effective partial ionic (orange) and electronic (blue) conductivities measured by impedance spectroscopy for composite cathodes with varying CAM volume fractions. Bottom: Corresponding calculated tortuosity factors for the same composite cathodes. Dashed lines serve as visual guides. b) Influence of varying CAM fractions on the specific charge capacity of SSB cathode composites. The specific charge is normalized to the cathode composite mass (top) and CAM mass (bottom). Reproduced with permission under the terms of the Creative Commons CC BY license 4.0.^[^ ^46^ ^]^ Copyright 2021, the Authors. Published by Electrochemical Society. c) Influence of grain boundary resistance (𝑅_GB_) on (top) effective ionic conductivity and normalized capacity, and (bottom) energy density of the composite cathode. Reproduced with permission under the terms of the Creative Commons CC BY license 4.0.^[^ ^105^ ^]^ Copyright 2023, the Authors. Published by Wiley‐VCH. d) Schematic depiction of composite cathodes composed of LFP‐bare and LFP‐nanosamples mixed with ceramic solid electrolyte. The LFP‐nanosample exhibits increased contact area between LFP and SE due to the reduced LFP particle size. Reproduced with permission.^[^ ^122^ ^]^ Copyright 2022, American Chemical Society. e) Schematic illustration of composite cathodes incorporating different Li_1.3_Al_0.3_Ti_1.7_(PO_4_)_3_ (LATP) particle‐size distributions. Reproduced with permission.^[^ ^123^ ^]^ Copyright 2024, Elsevier B.V.

A variation of the CAM‐to‐SE ratio leads to changes in microstructure which significantly influences the overall performance of composite cathodes in SSBs. To achieve a high energy density, the fraction of CAM should be maximized. Simulation studies^[^ ^45^, ^46^, ^104^, ^105^, ^106^ ^]^ indicate that a higher CAM ratio can enhance electronic conductivity and energy density but compromise ionic connectivity and increase SE‐phase tortuosity, thereby reducing rate capabilities. Conversely, elevated SE content ensures continuous ionic pathways but results in electronically isolated CAM particles, reduced active material utilization, and lower overall energy density. Minnmann et al.^[^ ^46^ ^]^ presented the ionic and electronic conductivity, as well as the tortuosity factor in a Li_6_PS_5_Cl–NCM622 system, as shown in **Figure** **5**a. As expected, with increasing CAM volume fraction, the electronic conductivity increases, and the ionic conductivity decreases. At around 42 vol% CAM, it shows a good balance between electronic and ionic conductivities, resulting in the highest CAM‐specific charge at all *C*‐rates (Figure 5b).

Oxide‐based electrolytes, particularly garnet‐type systems, such as LLZO, present unique challenges due to their inherent rigidity and the significant impact of grain boundaries.^[^ ^107^ ^]^ Recent 3D modeling^[^ ^105^ ^]^ on LCO cathodes with LLZO electrolytes has shown that grain boundary resistance significantly affects the ionic conductivity, capacity, and energy density of the cathode, as illustrated in Figure 5c. With the increase of grain boundary resistance, the maximum capacity shifts toward structures with lower LCO fractions. In cases of negligible grain boundary resistance, the highest energy is achieved at 70 vol% LCO. However, in practice, these systems often require relatively low CAM volume fraction (e.g., 30 vol%^[^ ^108^ ^]^) to ensure Li^+^ percolation throughout the cathode. To address these challenges, techniques such as optimizing sintering conditions, applying surface coatings, and doping with specific elements can be employed to reduce grain boundary resistance and enhance ionic conductivity.^[^ ^107^ ^]^ Additionally, optimizing electrode design, controlling particle size, and employing conductive additives can improve Li^+^ percolation and overall performance. This will be introduced in Sections 3.3 and 3.4.

Polymer‐based electrolytes, particularly PEO‐based systems, have demonstrated distinct advantages in terms of interfacial compatibility and mechanical flexibility. Typically, the solid polymer electrolyte content in PEO–lithium bis(trifluoromethanesulfonyl)imide (LiTFSI)‐based composite cathodes ranges from 10 to 30 wt%, significantly influencing electrochemical performance.^[^ ^109^, ^110^ ^]^ For example, in the LFP/PEO composite cathode system, using 60–70 wt% of LFP with the addition of nanoionic conductivity fillers can achieve stable electrochemical performance.^[^ ^111^ ^]^ However, the inherently low ionic conductivity of polymer electrolytes at low temperatures necessitates strategic cathode optimization. A common strategy involves adding inorganic nanofillers—either as crosslinking centers (e.g., SiO_2_^[^ ^112^ ^]^ and Al_2_O_3_^[^ ^113^ ^]^) to reduce polymer crystallinity and enhance chain mobility, or as fillers with high intrinsic ionic conductivity (e.g., LLZO^[^ ^114^ ^]^) to improve the overall ionic transport. Additionally, crosslinked polymers and modified polymers can enhance ionic conductivity while improving mechanical strength.^[^ ^115^ ^]^ For example, Fu et al.^[^ ^116^ ^]^ reported a dual‐salt PEO‐based polymer electrolyte utilizing a crosslinked network with tetraethylene glycol dimethyl ether (TEGDME), demonstrating a significantly improved ionic conductivity of 0.57 mS cm^−1^ at room temperature and a high lithium transference number of 0.79 due to crosslinking and intermolecular interactions. The polystyrene and poly(ethylene oxide) block copolymers (PS‐*b*‐PEO) with end‐group modification of PEO increased the free volume of PEO and altered the chain conformation, significantly enhancing ionic conductivity at room temperature.^[^ ^117^, ^118^ ^]^ To balance ionic and electronic conductivities, carbon‐based conductive additives (e.g., carbon black and carbon nanotubes (CNTs)) are introduced to establish electronic networks, particularly at lower CAM loadings. These additives, however, must be carefully moderated as they inherently occupy space without contributing to ionic conduction, thereby potentially increasing ionic tortuosity.^[^ ^111^, ^119^, ^120^ ^]^ In polymer‐based composite cathodes, a small amount of conductive filler can significantly boost electronic conductivity without harsh processing.^[^ ^110^ ^]^ For example, Orue et al.^[^ ^121^ ^]^ incorporated 8 wt% of CNTs into a PEO‐based LFP cathode, creating a co‐continuous conductive architecture that effectively facilitates fast Li‐ion transport while simultaneously enhancing electronic percolation.

The particle sizes of CAM and SE, along with their size ratios, critically influence ionic–electronic connectivity, interfacial contact area, and tortuosity, thereby impacting overall battery performance. Recent combined simulation and experimental studies highlight the importance of optimizing these parameters to enhance battery capacity, rate capability, and cycling stability.^[^ ^22^, ^103^, ^122^, ^123^, ^124^, ^125^, ^126^, ^127^, ^128^, ^129^, ^130^, ^131^, ^132^ ^]^ Cathode particle size influences both electron percolation network, and the intrinsic Li⁺ diffusion length within each particle. Larger CAM particles are beneficial for electronic connectivity; however, this benefit diminishes at high *C*‐rates. Small cathode particles offer shorter Li⁺ diffusion paths inside each particle, which is critical for high rate performance and ensuring complete utilization of the active material.^[^ ^124^, ^125^, ^126^ ^]^ It has been demonstrated that to achieve at least 83% of the theoretical specific capacity of a spherical CAM particle at a certain *C*‐rate, the particle size *L* should be designed based on the chemical diffusion coefficient *D*_Li_. This relationship can be expressed by the following equation^[^ ^133^ ^]^

(1) $$L\leq \sqrt{\frac{3\tilde{D_{\mathrm{Li}}}}{C-\mathrm{rate}}}$$

Benefits have been observed with small cathode particles. For example, Song et al.^[^ ^122^ ^]^ reported that nanoscale LFP particles dramatically improved cathode kinetics in a composite cathode (see Figure 5d); the nano‐LFP increased the contact area with the electrolyte and enhanced Li‐ion diffusion, leading to better capacity delivery at high rates. Similarly, experimental evidence from Kim et al.^[^ ^127^ ^]^ corroborates that nanostructured cathodes maintain higher capacities and exhibit improved cycling stability owing to enhanced ion transport at interfaces and within the active material. However, if the active material is finely divided into many sub‐micrometer particles, there is a risk of electronic isolation of some particles unless compensated by a robust conductive additive network.^[^ ^45^, ^128^, ^129^ ^]^ Moreover, the high specific surface area of small particles can lead to more severe interfacial chemical degradation during battery cycling.^[^ ^103^ ^]^

**Figure 6:**
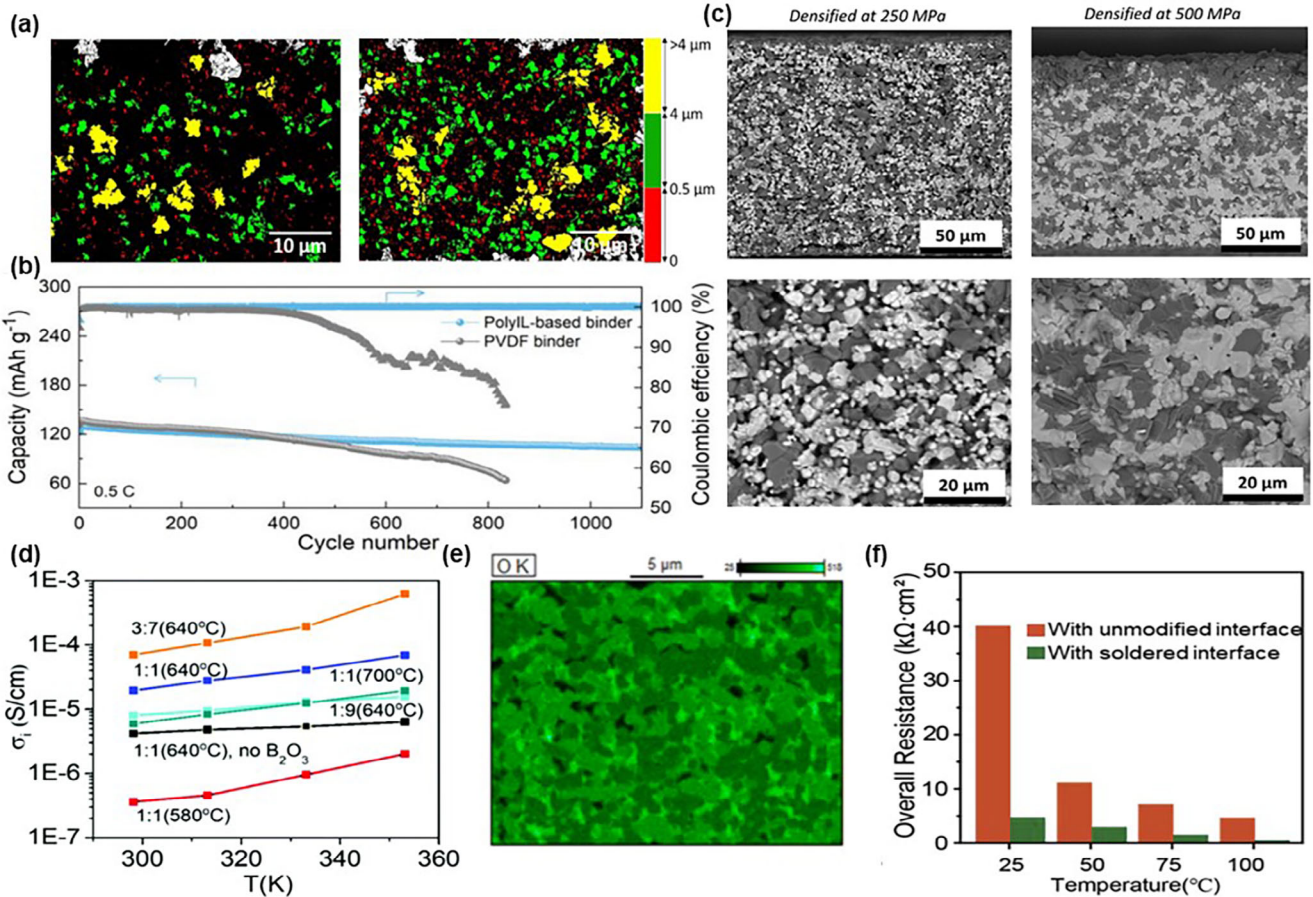
a) Particle size distribution of composite cathodes fabricated using magnetic stirring (left) and the ball milling method (right), with color codes indicating different components. Reproduced with permission under the terms of the Creative Commons CC BY license 4.0.^[^ ^111^ ^]^ Copyright 2022, the Authors. Published by Springer Nature. b) Cycling performance comparison utilizing PVDF binder versus poly(ionic liquid) (PolyIL)‐based binder. Reproduced with permission.^[^ ^137^ ^]^ Copyright 2025, Wiley‐VCH. c) Cross‐sectional SEM images of LCO–LLZTO composite cathodes densified at 80 °C under pressures of 250 and 500 MPa. Reproduced with permission under the terms of the Creative Commons CC BY license 4.0.^[^ ^138^ ^]^ Copyright 2023, the Authors. Published by American Chemical Society. d) Ionic conductivity of cathodes with and without B_2_O_3_ sintering additive. Reproduced with permission.^[^ ^141^ ^]^ Copyright 2021, Royal Society of Chemistry. e) Cross‐sectional energy‐dispersive X‐ray spectroscopy (EDS) oxygen map of the cathode layer. Reproduced with permission under the terms of the Creative Commons CC BY license 4.0.^[^ ^142^ ^]^ Copyright 2024, the Authors. Published by Royal Society of Chemistry. f) Overall resistance of the cells featuring either unmodified or soldered cathode interfaces. Reproduced with permission.^[^ ^143^ ^]^ Copyright 2020, Elsevier B.V.

Cathode performance is influenced not only by the size of the cathode particles but also by the size of SE particles. Smaller electrolyte particles generally promote better ionic percolation and lower tortuosity in the SE network because fine particles can pack into the interstices between larger active particles, creating continuous ion‐conduction pathways.^[^ ^45^, ^105^, ^123^, ^130^ ^]^ For example, in a composite cathode using Li_1.3_Al_0.3_Ti_1.7_(PO_4_)_3_ (LATP) solid electrolyte, as shown in **Figure** **6**e, a smaller particle‐size distribution enabled a more uniform Li⁺ transfer network, enhancing the electrochemical stability and rate performance of SSBs.^[^ ^123^ ^]^ However, extremely fine particles introduce numerous grain boundaries and particle/particle interfaces, which can raise the internal ionic resistance.^[^ ^105^ ^]^

In general, optimal particle size ratio (*λ* = *D*_CAM_/*D*_SE_) should be balanced to ensure effective percolation in the cathode.^[^ ^45^, ^105^, ^131^, ^132^ ^]^ Mismatches in cathode/electrolyte particle sizes can create void spaces and poor contacts in the composite. A higher ratio (*λ*) tends to form open channels for the electrolyte phase, lowering ionic path tortuosity. Simulation from Clausnitzer et al.^[^ ^105^ ^]^ indicates that high *λ* yields lower ionic tortuosity in the electrolyte phase—beneficial for ion transport. Experimentally, Shi et al.^[^ ^45^ ^]^ found that increasing the CAM:SE size ratio in NCM cathodes with sulfide SE enabled much higher active material loadings without creating tortuous ion paths. On the other hand, if *λ* is too high, large cathode particles lengthen the Li⁺ diffusion distance, and very fine SE particles may introduce the aforementioned grain boundary issues. In summary, there is a “sweet spot” in particle sizes where the electrolyte network is percolating and minimally resistive.

The “optimal” CAM:SE ratio is not universal; it depends on the SE family, grain‐boundary resistance in oxides, the particle size ratio, and the morphology of conductive additives. Aforementioned literature values are useful starting points. Composition optimization should be co‐designed with interface engineering and electrode architecture/processing optimization to maintain balanced ion/electron transport and achieve durable cycling performance even in thick, high‐loading composite cathodes.

### 3.3 Mitigating Interfacial Challenges during Cathode Processing

Due to the solid–solid nature of composite cathodes, where the CAMs, SEs, and conducting agents are physically mixed, mixing process critically determine the microstructure and connectivity of the ionic and electronic pathways. In polymer‐based SSBs, polymer SEs exhibit viscoelastic behavior, serving dual roles as ion‐conductive media and mechanical binders. This dual functionality enhances wetting and conformal contact with the surface of active materials. High‐energy mixing methods, such as planetary mixing, ball milling, or speed mixing, are employed to break up particle agglomerates and promote uniform distribution.^[^ ^134^ ^]^ Notably, the wet ball milling method assists in pulverizing the particles and improving homogeneity of the electrode compared to conventional magnetic stirring methods, resulting in enhanced electrochemical performance with the composite cathode mixture consisting of LFP, Super P, PEO, LiTFSI, and acetonitrile.^[^ ^111^ ^]^ As shown in Figure 6a, the cathode produced using the ball milling process in the right image is more homogeneous compared to the cathode produced using magnetic stirring in the left image. Upon drying, the polymer matrix embeds the active material particles, creating continuous interfacial contact that accommodates volume changes during cycling. As a result, polymer‐based composite cathodes generally exhibit lower interfacial resistance and improved mechanical cohesion at the CAM/SE interface, even under low or no external pressure.^[^ ^135^, ^136^ ^]^ Compared to conventional poly(vinylidene fluoride) (PVDF) binder, poly(ionic liquid) that simultaneously serves as a matrix of polymer SE and a cathode binder significantly improves the interface between CAMs and SE, exhibiting stable cyclability over 1100 cycles (Figure 6b).^[^ ^137^ ^]^

In contrast, oxide‐based SEs are brittle, nondeformable ceramics with limited ability to conform to the CAM surface. As a result, mixing and microstructural engineering become essential to overcome interfacial voids and contact loss. Therefore, oxide‐based composite cathode slurry typically requires CAM particles, SE powders, and a binder such as PVDF, which are dispersed in a solvent like *N*‐methyl‐2‐pyrrolidone (NMP), ethanol, or isopropanol. Particularly, Ye et al. demonstrated the feasibility of aqueous processing for LCO–Li_6.6_La_3_Zr_1.6_Ta_0.4_O_12_ (LLZTO) composite cathodes by using methylcellulose as a binder and employing polyethylene glycol and glycerol as plasticizers.^[^ ^138^ ^]^ Due to the rigid nature of both CAM and oxide SE, even well‐mixed composite cathodes often require postprocessing steps—such as calendaring or hot pressing—to physically enforce contact at the interface. The effect of pressing for interfaces between cathode particles and SEs has been validated including uniform pore size distribution, reduced porosity, densification, and enhanced ionic/electronic conductivity.^[^ ^139^, ^140^ ^]^ For example, LCO–LLZTO composite cathodes, prepared using the slurry casting method, were compacted under varying pressures to assess the effects of pressing, implying insufficient pressure results in poor contact (Figure 6c).^[^ ^138^ ^]^

To further reduce the interface resistance between CAM and oxide‐based SE, a co‐sintering process is a promising approach that achieves intimate contact between these components. However, oxide‐based SE typically requires high sintering temperatures, which complicates the co‐sintering process with other cathode components. To address these challenges, researchers have explored sintering additives and novel sintering techniques. For instance, Han et al. reported that a B_2_O_3_ sintering additive effectively lowers the sintering temperature of NASICON‐type LATP solid‐state electrolytes from 900 to 750 °C.^[^ ^141^ ^]^ Incorporating a sintering aid allows co‐sintering at 640 °C, densifying the composite cathode layer and enhancing the ionic conductivity to ≈1.9 × 10^−5^ S cm^−1^ (Figure 6d). Using Li_3_BO_3_ as a sintering additive for Li─Sb─O‐type SEs reduces the cathode sintering temperature to 750 °C, resulting in a high relative density of the cathode layer of 93% and an enhanced contact between LCO particles and SEs (Figure 6e).^[^ ^142^ ^]^ Furthermore, employing a fast microwave soldering technique improves efficiency and minimizes the side effects of long sintering time, significantly improved interface between the cathode particles and solid‐state electrolytes (Figure 6f).^[^ ^143^ ^]^

The intricate interplay between material composition, mixing techniques, and postprocessing methods is pivotal in advancing composite cathode technologies for SSBs. By refining these processes, researchers can overcome inherent challenges such as interfacial resistance and mechanical cohesion, ultimately unlocking the full potential of SSBs. The strategic use of polymer matrices, pressing, sintering additives, and innovative processing techniques not only enhances ionic and electronic conductivity but also ensures the structural integrity and longevity of the battery system. From a practical standpoint, process selection must consider not only interfacial performance but also cost, cycle time, and scalability. Taking manufacturability constraints—cost, throughput, yield, and safety (solvent handling and moisture sensitivity)—into account, processes that reduce temperature, pressure, solvent use, and sintering dwell time while preserving robust solid–solid contact should be prioritized. Specifically, dry processing—including polymer fibrillation, dry‐spray coating, direct compressing—eliminates solvent usage and drying operations, which can significantly lower costs related to solvent handling and drying while reducing environmental footprint.^[^ ^144^, ^145^ ^]^ By avoiding slurry‐induced segregation and solvent‐mediated interfacial reactions, its potential is considerable.

### 3.4 Cathode Structural Design

Despite careful optimization of composite composition and the use of advanced mixing and densification techniques, conventional homogeneous cathode architectures in SSBs often struggle to satisfy the competing requirements of high energy density and fast kinetics, especially in thick electrodes under high‐rate conditions. Recent perspectives have spotlighted the need for specialized cathode architectural engineering to overcome this bottleneck.

**Figure 7:**
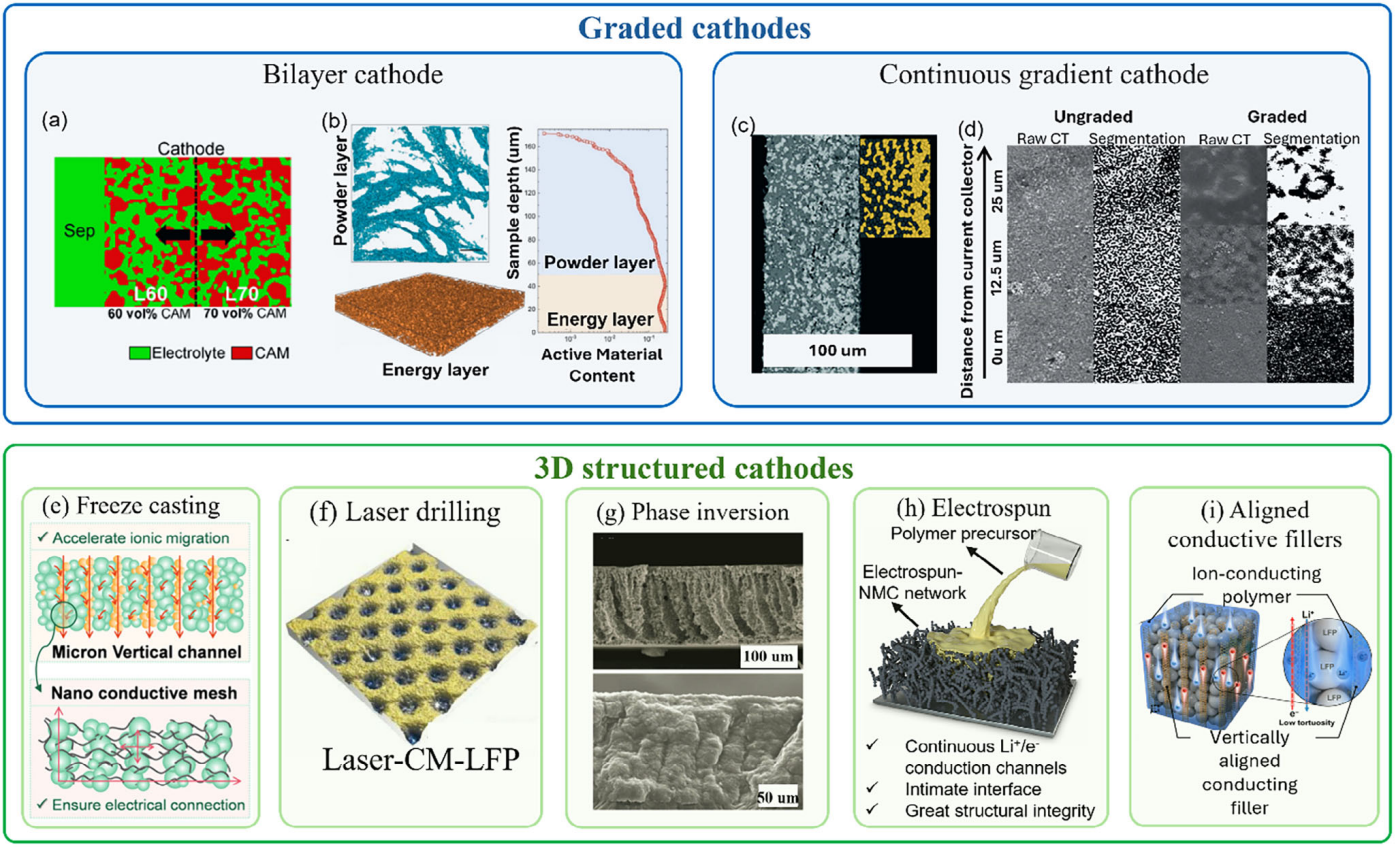
a) Cross‐sectional view of simulated layered cathode microstructures for SSBs, featuring a fixed total thickness and two layers with 60 vol% CAM near the separator and 70 vol% CAM near the current collector. Reproduced with permission under the terms of the Creative Commons CC BY license 4.0.^[^ ^102^ ^]^ Copyright 2024, the Authors. Published by Wiley‐VCH. b) 3D top‐view representations (650 × 650 µm^2^) of the power and energy layers in the two‐layered cathode structure, along with the variation of composite cathode fraction (active material content) as a function of total cathode thickness. Reproduced by permission.^[^ ^147^ ^]^ Copyright 2023, Elsevier Ltd. c) Microstructure of the gradient cathode formed by sequential layer casting, enabling the design of opposing concentration gradients for the active material and electrolyte across the cathode thickness. Reproduced by permission.^[^ ^152^ ^]^ Copyright 2022, The Royal Society of Chemistry. d) Characterization of graded and ungraded composite cathodes using X‐ray computed tomography (CT), showing three raw CT slices and corresponding segmentations at varying distances from the current collector. Reproduced with permission under the terms of the Creative Commons CC BY license 4.0.^[^ ^153^ ^]^ Copyright 2024, the Authors. Published by The Royal Society of Chemistry. e) Schematic diagram illustrating the fabrication of vascularized electrodes using the freeze‐casting technique. Reproduced by permission.^[^ ^158^ ^]^ Copyright 2024, American Chemical Society. f) 3D reconstructed image of laser‐drilled composite cathode. Reproduced by permission.^[^ ^155^ ^]^ Copyright 2021, Elsevier B.V. g) Cross‐sectional SEM image of a low‐tortuosity LiFePO_4_ electrode and corresponding composite electrode, fabricated via water phase inversion. Reproduced by permission.^[^ ^160^ ^]^ Copyright 2022, Elsevier B.V. h) Schematic illustration of the fabrication process for the composite electrospun‐NCM cathode. Reproduced by permission.^[^ ^162^ ^]^ Copyright 2022, Elsevier B.V. i) Structure of the cathode and schematic illustration of the conduction synergy mechanism in the filler‐aligned structured thick (FAST) cathode. Reproduced by permission.^[^ ^161^ ^]^ Copyright 2025, American Association for the Advancement of Science.

In conventional homogeneous cathodes, ionic‐transport resistance tends to accumulate toward the current‐collector side, while electronic resistance builds up toward the separator side. This results in nonuniform reaction distribution and under‐utilization of active material at high rates. Graded cathode architecture has emerged as a compelling solution to address these issues. A gradient structure intentionally varies composition or particle characteristics across the cathode thickness to achieve high electronic conductivity near the current collector and high ionic conductivity near the SE separator. This compensates for these heterogeneities in local resistance, ensuring favorable pathways for both ions and electrons throughout the cathode.^[^ ^146^, ^147^, ^148^, ^149^ ^]^ Simulations and modeling have quantitatively demonstrated the benefits of graded cathodes.^[^ ^48^, ^102^, ^133^, ^150^, ^151^ ^]^ Usiskin and Maier^[^ ^133^ ^]^ showed that distributing more SE toward the electrolyte side and more conductive/carbon phase toward the current‐collector side can dramatically reduce polarization during fast charge. Similarly, Clausnitzer et al.^[^ ^102^ ^]^ recently reported that a two‐layer gradient model (**Figure** **7**a) improves the balance between electronic and ionic networks, resulting in higher usable capacity at 5 C discharge. Experimentally, a practical approach involves fabricating a bilayer cathode, with each layer optimized for either electronic or ionic conduction. Polizos et al.^[^ ^147^ ^]^ created a two‐layered cathode consisting of a bottom “energy layer” (densified NCM622 composite for high volumetric energy) and a top “power layer” (high‐porosity NCM622 with vertically aligned walls via ice templating) within a single electrode (Figure 7b). This design achieved more than double the capacity values compared to nonstructured cathodes in the PEO‐based SE system. Beyond the bilayer structure, various methods have been applied to create continuous gradient cathodes. Rosen et al.^[^ ^152^ ^]^ constructed a centration gradient LCO‐LLZO cathode (Figure 7c) using a sequential layer‐casting method, achieving a cathode with an areal capacity 2.8 mAh cm^−2^ utilizing 99% of the theoretical capacity. Tudball et al.^[^ ^153^ ^]^ manufactured gradient composite cathodes (LFP–PEO) using spray deposition (Figure 7d), which showed ten times lower resistance and excellent rate performance compared to the uniform cathodes. All experimental and practical results evidence that graded designs, achieved by varying composition or microstructure across the electrode, represent a promising engineering solution for high loading, high capacity, and high‐rate capability cathodes.

Beyond gradient design, researchers are exploring diverse methods to build low tortuosity in composite cathodes by 3D structural design. These designs feature vertically oriented channels,^[^ ^101^, ^154^, ^155^, ^156^, ^157^, ^158^ ^]^ interconnected porous networks,^[^ ^159^, ^160^ ^]^ and aligned conductive fillers,^[^ ^158^, ^161^ ^]^ all of which enhance both ionic and electronic pathways. Several innovative fabrication methods have been investigated to realize effective 3D composite cathode structures. Freeze casting (ice templating) has been widely adopted to produce vertically aligned porous structures, enhancing electrolyte infiltration and ion transport.^[^ ^147^, ^154^, ^156^, ^157^, ^158^, ^159^ ^]^ Recently, Song et al.^[^ ^158^ ^]^ enabled the construction of vertically oriented low‐tortuosity channels, significantly enhancing ion transport pathways within thick composite electrodes for SSBs. Their advanced cathode design, termed a “vascularized electrode” as shown in Figure 7e, effectively decoupled ionic and electronic transports, achieving simultaneous high power density (>1600 W kg^−1^) and ultrahigh areal capacities (≈5.25 mAh cm^−^^2^). Despite these notable advantages, freeze casting requires precise thermal control and faces scalability issues due to the complexity of maintaining uniform channel alignment over large areas. Laser technology is another effective technique that provides precisely controlled, uniform vertical microchannels.^[^ ^101^, ^155^ ^]^ Wu et al.^[^ ^155^ ^]^ created uniform vertical channels within thick LFP electrodes using laser technology, as the structure shown in Figure 7f, resulting in cathodes with significantly enhanced cycling stability and high areal capacity (≈5.3 mAh cm^−^^2^ at 0.5 C), attributed to rapid ion diffusion through the engineered vertical microchannels. However, laser drilling is inherently energy intensive, poses challenges for scale‐up, and can introduce thermal‐induced defects that affect long‐term mechanical stability. Phase inversion has emerged as a scalable and versatile method for introducing vertically aligned channels into thick electrodes. Wang et al. reported composite cathodes with vertically structured channels created via phase inversion.^[^ ^160^ ^]^ Their advanced composite cathode design incorporates these structured channels filled via in situ polymerization (Figure 7g), resulting in high areal capacities up to 5 mAh cm^−^^2^ and excellent cycling stability, maintaining 94% capacity retention over 150 cycles at 0.5 C. While promising, the phase inversion method requires precise formulation and careful control over solvent/nonsolvent interactions to ensure uniform channel structures and mechanical integrity at larger scales. Electrospinning allows the integration of cathode active materials, polymer electrolytes, and conductive fibers into a finely interconnected, flexible, and porous network. Zhang and co‐workers.^[^ ^162^ ^]^ developed a 3D cathode using crosslinked electrospun NCM integrated with PEO and carbon nanofibers (Figure 7h), achieving high specific capacities (154 mAh g^−1^ at 0.1 C) and superior cycling stability. However, electrospinning typically involves slow throughput and complexity in producing thick and mechanically robust electrodes. Beyond 3D design on CAM or SE, vertically aligned electron‐conducting CNTs are designed to be uniformly distributed within LFP cathodes to create a low‐tortuosity electron/ion transport path.^[^ ^161^ ^]^ The resulting filler‐aligned structured thick (FAST) cathode (Figure 7i) demonstrated high capacity retention (148 mAh g^−1^ over 100 cycles) and exceptional power density (1600 W kg^−1^), far surpassing conventional slurry‐cast electrodes. Nonetheless, achieving uniform distribution and alignment of CNTs remains challenging at scale.

Despite these advancements, several challenges remain to be addressed. Large‐scale manufacturing of complex 3D cathode structures is technically challenging due to the precision required for processes such as freeze casting or laser drilling. In addition, maintaining mechanical integrity during operation is critical, as the introduction of engineered porosity and channels can potentially compromise structural stability. In summary, the 3D‐structured cathode architecture is a highly promising approach to overcoming charge transport limitations in conventional SSB cathodes. Continued optimization and innovation in fabrication processes will be needed for converting these promising lab‐scale achievements into large‐scale manufacturing.

## 4 Conclusions and Perspectives

In conclusion, the battery research community has been actively developing novel materials, structures, and synthesis approaches to address the challenges in SSB cathodes, which has achieved significant improvement. However, there still exist significant challenges, requiring co‐optimization of different aspects during battery engineering. To realize SSBs’ potential in high energy density, high‐performance cathodes are necessary, which should exhibit high areal loading, high active material content, high electronic and ionic conductivity, and long cycle life. CAMs and catholyte need to be designed and processed to increase contact between these two components while maximizing the CAM content. Particle properties, such as particle size, morphology, and surface area need to be optimized and facilitate subsequent cathode fabrication process. As conventional slurry‐based processed cathodes exhibit high porosity resulted from solvent removal, new electrode processing methods are needed to produce dense cathodes. Dry processing poses many advantages in fabricating thick electrodes with excellent mechanical integrity, uniform binder distribution, and low porosity and can be applied to fabricate SSB cathodes. Tailored cathode architectures such as gradient cathode structure could enhance active material utilization by optimizing electronic and ionic conductivities.

Leveraging advanced material and cell characterization techniques, along with electrochemical modeling, enables real‐time observation and deeper understanding of the dynamic changes in cathode chemistries, microstructures, and interfaces during cycling. These insights are crucial for elucidating how such changes influence charge‐transfer processes, such as ion insertion and extraction, concentration gradients, transport pathways, and transfer kinetics. This integrated approach fosters a more comprehensive understanding of cathode electrochemical performance and degradation mechanisms, ultimately guiding material design and process optimization. Some key modeling strategies include^[^ ^6^ ^]^ 1) electrode kinetics models that depict charge‐transfer reactions, electrode reaction mechanisms, activation polarization, and mass transport; 2) solid electrolyte–electrode interface models that describe interface reactions, interphase layer formation, kinetics of charge transfer, ion transport, and thermal and mechanical effects; 3) multiphysics coupling models that integrate interacting phenomena related to ion transport, thermal effects, and mechanical stress; and 4) phase field model that simulates the evolution of phases and interfaces within materials.

Calibrating these models using advanced in situ and operando characterization techniques,^[^ ^71^, ^163^ ^]^ which are nondestructive and provide real‐time information, greatly enhances their accuracy and adaptability. Numerous in situ and operando tools based on synchrotron X‐ray techniques, such as X‐ray diffraction, pair distribution function analysis,^[^ ^163^, ^164^, ^165^ ^]^ X‐ray spectroscopy methods^[^ ^166^, ^167^, ^168^ ^]^ (e.g., X‐ray absorption spectroscopy, X‐ray emission spectroscopy, and ambient pressure X‐ray photoelectron spectroscopy), and X‐ray imaging^[^ ^169^, ^170^ ^]^ (e.g., microtomography and ptychography) offer excellent spatial, temporal, and energy resolution and are particularly valuable for studying interfacial phenomena. In particular, these techniques resolve transition‐metal oxidation states and coordination changes, revealing CAM/SE interfacial reactions and structural instabilities that lead to microcracks and pore formation—microstructural degradation directly linked to failure modes. Additional techniques include environmental electron microscopy^[^ ^171^ ^]^ (e.g., scanning electron microscopy and transmission electron microscopy), Raman spectroscopy, and infrared spectroscopy.^[^ ^172^, ^173^ ^]^ Environmental electron microscopy can visualize and quantify secondary phases formed by interfacial reactions and assess their contribution to grain‐boundary resistance, while Raman/IR spectroscopy probes how reductions in polymer crystallinity and changes in chain conformation in polymer SEs translate into improved ionic conductivity and interfacial contact. These advanced tools facilitate the identification of nonequilibrium state materials or phases, as well as the rapid but transient process occurring during chemical/electrochemical reactions. Such capabilities are invaluable for diagnosing the failure modes of SSBs and guiding performance improvements.

Given the limitations of individual modeling strategies and characterization techniques, we emphasize the importance of combining multiple models with a multimodal characterization approach. For example, combining X‐ray microscopy and spectroscopy can provide detailed insights into both morphological and chemical structures.^[^ ^170^ ^]^ Furthermore, the recent application of machine learning to analyze large parameter spaces and manage multitask, multiobjective simulations are beneficial.

## Conflict of Interest

The authors declare no conflict of interest.
